# Effects of a Single Opioid Dose on Gastrointestinal Motility in Rabbits (*Oryctolagus cuniculus*): Comparisons among Morphine, Butorphanol, and Tramadol

**DOI:** 10.3390/vetsci9010028

**Published:** 2022-01-14

**Authors:** Hélène Deflers, Frédéric Gandar, Géraldine Bolen, Johann Detilleux, Charlotte Sandersen, Didier Marlier

**Affiliations:** 1 Exotic Pet Department, Veterinary Clinic Alvetia, F57100 Thionville, France; helene.deflers@gmail.com (H.D.); fgandar@hotmail.com (F.G.); 2 Diagnostic Imaging Section, Department of Clinical Sciences of Companion Animals and Equine, Fundamental and Applied Research for Animals & Health (FARAH), Faculty of Veterinary Medicine, University of Liège, B4000 Liège, Belgium; gbolen@uliege.be; 3 Unit of Animal Breeding, Department of Veterinary Management of Animal Resources, FARAH, Faculty of Veterinary Medicine, University of Liège, B4000 Liège, Belgium; jdetilleux@uliege.be; 4 Anaesthesiology Section, Department of Clinical Sciences of Companion Animals and Equine, FARAH, Faculty of Veterinary Medicine, University of Liège, B4000 Liège, Belgium; Charlotte.Sandersen@uliege.be; 5 Clinic for Birds, Rabbits and Rodents, Department of Clinical Sciences of Companion Animals and Equine, FARAH, Faculty of Veterinary Medicine, University of Liège, B4000 Liège, Belgium

**Keywords:** *Oryctolagus cuniculus*, butorphanol, morphine, tramadol, gastrointestinal motility

## Abstract

The aim of this study was to evaluate and compare the effects of single doses of butorphanol, morphine, and tramadol on gastrointestinal motility in rabbits (*Oryctolagus cuniculus*) using non-invasive imaging methods, such as radiographic barium follow through and ultrasonographic contraction counts. Time-lapse radiographic and ultrasound examinations were performed before and after a single intramuscular dose of 5 mg kg^−1^ butorphanol, 10 mg kg^−1^ morphine, or 10 mg kg^−1^ tramadol. Pyloric and duodenal contraction counts by ultrasonography and radiographic repletion scores for the stomach and caecum were analysed using a mixed linear model. No significant effect was noted on ultrasound examinations of pyloric and duodenal contractions after administration of an opioid treatment. Morphine had a significant effect on the stomach and the caecum repletion scores, whereas butorphanol had a significant effect only on the caecum repletion score. Tramadol had no significant effect on the stomach or caecum repletion scores. The present findings suggest that a single dose of 5 mg kg^−1^ butorphanol or 10 mg kg^−1^ morphine temporarily slows gastrointestinal transit in healthy rabbits, preventing physiological progression of the alimentary bolus without the induction of ileus. In contrast, a single dose of 10 mg kg^−1^ tramadol has no such effects.

## 1. Introduction

Opioids are known to produce gastrointestinal side effects, such as constipation, in humans [1]. Data about rabbits, however, remain scarce and even controversial. To the authors’ knowledge, only the effects of buprenorphine on gastrointestinal motility have been studied so far. While some studies reported that buprenorphine decreased faecal output without other clinical consequences after several administrations over two days [2,3], another study showed that a single intramuscular (IM) administration of buprenorphine had no effect on gastrointestinal motility in rabbits, i.e., the alimentary bolus smoothly continued its progression [4]. However, the protocols differed among studies; the former focused on effects related to several administrations, whereas the latter studied the temporary effect of a single high dose of 0.1 mg kg^−1^.

In rabbits, gastrointestinal complications are common following an anaesthesia [5]. Given gastrointestinal side effects are reported with opioid use in small mammals, the ultimate complication might well be a postoperative ileus [6]. Ileus in rabbits is a serious condition because gastrointestinal hypomotility can lead to dysbiosis [7] and consequently to gas accumulation within the intestines and/or stomach, from which it cannot escape because of the well-developed cardia in this species [8]. This is a life-threatening condition because of the ischaemic consequences [7]. Indeed, gastrointestinal disease resulting in ischaemia may lead to the loss of intestinal integrity and subsequent bacterial translocation [9]. In rats, morphine-induced alterations in intestinal transit was proven as cause of bacterial translocation [10]. For these reasons, authors believe that veterinary surgeons who are not familiar with rabbit medicine may be less likely to administer opioids to rabbits than to conventional domestic mammals; consequently, analgesic management for these species may be not optimal.

On the basis of a previous study performed in horses, which are also hindgut fermenters [11], we hypothesised that morphine and butorphanol, but not tramadol, slow the gastrointestinal transit in rabbits. To test this hypothesis, we conducted the present study that aimed to evaluate and compare the effects of a single dose of three different opioids, namely morphine, butorphanol, and tramadol, on gastrointestinal transit in healthy young male rabbits using a non-invasive imaging protocol [4].

## 2. Materials and Methods

### 2.1. Animals and Experimental Protocol

New Zealand white rabbits (*n* = 45; mean weight ± standard deviation: 2.59 ± 0.40 kg), obtained from the Centre d’Economie Rurale de Marloie (CER, B6900 Marloie, Belgium), were included in the present study. The 45 animals were 3 months old and were specific pathogen-free. The animals were housed in pens (width × depth × height: 1.5 × 1.4 × 1.5 m) containing wood shavings as bedding material in groups of 5, as required by the ethics committee. They were randomly allocated to these lots of five at the time of arrival, using the standard = RAND function in Microsoft Excel. Pens had a resting/hiding platform which increased their surface with additional 0.9 m². Environmental enrichment was provided with hay distributed in several places and wooden sticks. Hay and a commercial extruded rabbit diet, comprising 14% proteins, 3.0% fat, and 30 % crude fibres, crude ash 7.5%, calcium 0.8%, and phosphorus 0.6%, were provided ad libitum. Composition of the extruded rabbit diet was derivatives of vegetable origin (timothy, grasses, and herbs 10%), vegetables (carrot 4%), vegetable protein extracts, seeds (linseed 2%), minerals, fructo-oligosaccharides (0.3%), calendula, and yucca. Water was constantly available. Temperature of the rabbitries was 20 °C and light was provided during 12 h.

An acclimatisation period of 7 days preceded all the experiments. During this period, the rabbits were regularly handled, so that they got used to the recumbent position for subsequent imaging examinations.

The 45 rabbits were split into equal groups, with 15 rabbits in each opioid group designated to receive one of 3 molecules: morphine, butorphanol, or tramadol. This protocol was designed to evaluate the gastrointestinal adverse effects of opioids in rabbits. As requested by the ethics committee, termination criteria were defined to stop experiments if signs of ileus or pain were observed.

### 2.2. Nasogastric Tube Placement

The day before radiographic examinations, a nasogastric tube (feeding tube CH6 500 mm; Dahlhausen and Co, 50996 Köln, Germany) was placed in each rabbit under sedation with intravenous (IV) medetomidine 150 µg kg^−1^ (Narco Start; Kela Veterinaria, B2320 Hoogstraten, Belgium) administered in the posterior marginal ear vein. Reversal was achieved with 375 µg kg^−1^ of intramuscular (IM) atipamezole (Narco Stop; Kela Veterinaria, B2320 Hoogstraten, Belgium), administered in the lumbar region. Before the procedure, the tube was measured. The tube length to introduce corresponded to the distance comprised between the animal nose and its 13th rib. Prior to nasal tube introduction, the nostril was applied topical lidocaine 10% (Xylocaïne 10% Spray; AstraZeneca, B1702 Dilbeek, Belgium). The tube was lubricated to ease the insertion into the medial aspect of the nostril and pushed until the pharynx was reached. The rabbit head was rostrally tilted to facilitate pharyngeal intromission of the tube. Confirmation of the correct placement was achieved thanks to the aspiration of gastric content. After good placement confirmation, the tube was attached with skin stiches on the skin close to the external nasal edge and on the top of the head. During the entire procedure, the animals were monitored with a pulse oximeter (Impact III; Vetronic Services, TQ12 5NF, Abbotskerswell, UK). Nasogastric tubes were removed from rabbit nostrils one hour after the opioid injection.

### 2.3. Radiographic and Ultrasound Examinations

Two sets of serial radiographic and two sets of serial ultrasound examinations were performed. Administration of a 10 mL kg^−1^ barium meal via the nasogastric tube was performed for radiographic series only, using the same method and schedule as previously described [4].

Baseline radiographic examinations were performed without any treatment (control) at 0, 0.25, 0.5, 1, 3, 6, 12, and 24 h after administration of barium meal. Examinations were repeated 1 week later. Intramuscular injection of a single dose of 5 mg kg^−1^ butorphanol (Butomidor, Ecuphar, B8020 Oostkamp Belgium), 10 mg kg^−1^ morphine (Morphine HCl, Sterop, B1070 Brussels, Belgium), or 10 mg kg^−1^ tramadol (Tramadol EG, Eurogenerics, B1020 Brussels, Belgium), according to the treatment group, was performed 5 min before barium meal administration. Injection was administered in the lumbar region.

One board-certified radiologist, unaware of the treatment protocol, used an adaptation of the Kobayashi and Horii stomach repletion score to analyse the stomach and caecum barium follow through repletion times [12], as previously described [4].

Baseline ultrasound examinations were performed without any treatment (control) at 0, 0.5, 1, 3, 6, 12, and 24 h. Examinations were repeated 1 week later. Intramuscular administration of 1 of the 3 opioids mentioned before, 5 min before each timing examination. In each ultrasound series, pyloric and duodenal contractions were counted in real time during a 2 min period by the same board-certified radiologist who performed the radiographic examinations.

Sets of serial ultrasound examinations were performed within 2 days because of the 24 h imaging timing (T0–T24 h), that corresponded to the 1st and 2nd weekday, then rabbits rested for 1 day after the procedure. After this resting day, animals underwent the nasogastric tube placement on the 4th day, and radiographic session started the day after and lasted 2 days (T0 to T24 h), i.e., the 5th and 6th day of the week.

### 2.4. Euthanasia

All rabbits were humanely euthanised after the second radiographic and ultrasound examinations were complete, as required by the local ethics committee, i.e., once all the respective sets of serial radiographic and ultrasound examinations were completed. Medetomidine 100 µg kg^−1^ (Narco Start; Kela Veterinaria, B2320 Hoogstraten, Belgium), ketamine 5 mg kg^−1^ (Nimatek; Eurovet Animal Health, 5531 AE Bladel, The Netherlands), and butorphanol 100 µg kg^−1^ (Butomidor; Ecuphar, B8020 Oostkamp, Belgium) were intramuscularly administered as premedication. Once the righting reflex and immobility were observed, the response to ear and forelimb pinching was assessed. If there was no response, a mixture of embutramide, mebezonium iodide, and tetracaine (1 mL per animal; T 61; MSD Animal Health, B1200 Brussels, Belgium) was directly administered into the heart of the animals. Death was confirmed after heartbeat and breathing ceased, along with pronounced pupil mydriasis and cyanosis of the buccal mucosa.

### 2.5. Statistical Analysis

The number of rabbits was chosen according to another close protocol [4], where 15 rabbits were needed in order to achieve a size effect of 0.64 considering a 1-tailed z-test with a power of 0.80 and an alpha error of 0.05, assuming a normal distribution.

Means values for all data are provided with the corresponding standard error of the mean (SEM). All statistical analyses were carried out using the SAS software (SAS 9.1, SAS Institute, Cary, NC, USA) and the significance threshold was set at 5%. Firstly, the assumption of normality for the stomach and caecum radiographic repletion scores (RRS) and the number of pyloric and duodenal contractions was assessed with the Shapiro–Wilk and Kolmogorov–Smirnov tests. In case of violation of this assumption, non-normality was reduced by a Box–Cox power transformation. Four separated linear mixed repeated models were used to determine whether hours of measurement after injection (0, 0.25, 0.5, 1, 3, 6, 12, 24), group (before or after injection), opioid drug (morphine, butorphanol, tramadol), and lot within treatment (1 to 9), and all interaction terms had an effect on the measurements. For each rabbit, time was assumed to be normally distributed with a null mean and (co)variance with an auto-regressive structure type 1. When the null hypothesis of no differences among means was rejected by the models, post hoc multiple comparison tests were used to analyse specific aspects between group means and *p*-values were adjusted to control the family-wise error rate (Bonferroni method). A *p*-value of <0.05 was considered statistically significant. Results from the linear mixed repeated models are presented as least square means because this guarantees best adjustment to the experimental data.

## 3. Results

One rabbit from the morphine cohort was excluded from the study because it displayed pronounced apathy and anorexia following the baseline radiographic examinations before any treatment was administered. As these were termination criteria, the rabbit had to be euthanized. Necropsy did not reveal any abnormal gross lesions. Consequently, radiographic and ultrasound data were obtained for 44 rabbits. No side effects, such as sedation after butorphanol or tramadol administration, were observed. However, morphine administration resulted in sedation and altered breathing pattern with more profound breathing instead of shallow breathing. The affected rabbits remained sedated for 3 h after administration of morphine. Except from the rabbit excluded from the morphine group, none of the treated animals developed overt ileus. All rabbits except the ones who were sedated by morphine ate immediately after the examinations.

**Figure 1:**
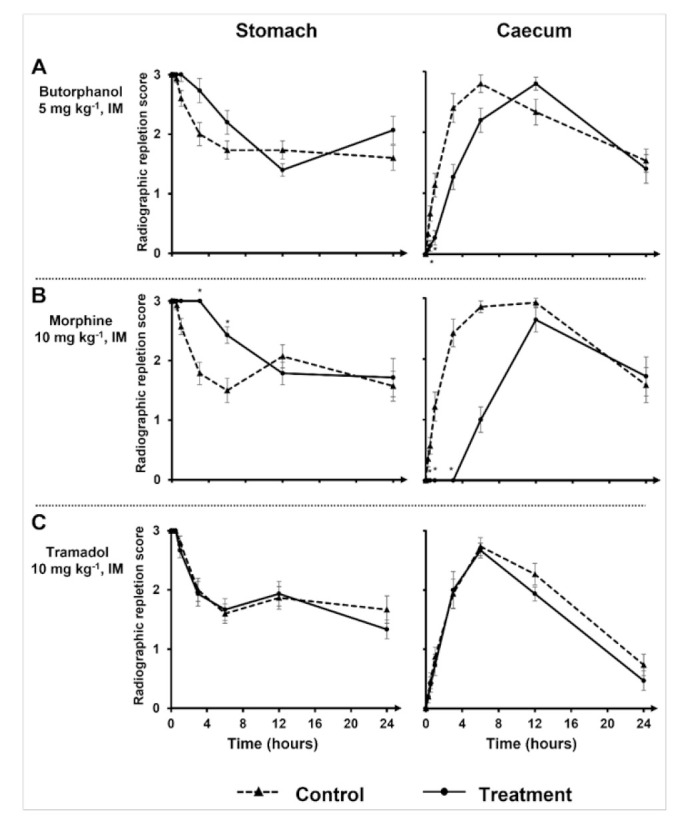
Radiographic repletion scores for the stomach and caecum as a function of time, according to the different treatments used (butorphanol 5 mg kg^−1^, IM; morphine 10 mg kg^−1^, IM; tramadol 10 mg kg^−1^, IM). Radiographic repletion scores for the stomach and caecum (Mean ± standard error of the mean (SEM)) as a function of time (hours), obtained via a barium follow through examination without (control) and after an intramuscular injection of 5 mg kg^−1^ of butorphanol (*n* = 15) (**A**), 10 mg kg^−1^ of morphine (*n* = 14) (**B**), and 10 mg kg^−1^ of tramadol (*n* = 15) (**C**) in rabbits. T0 corresponds to administration of the barium meal. Injection of the opioid was performed 5 min before barium meal administration. The stomach score was assigned as follows: 0—no barium; 1—small amount of barium; 2—medium quantity of barium, heterogeneous repartition of contrast; 3—large amount of barium, homogeneous repartition of contrast or identical to the first radiograph taken at T0 min, when barium has just reached the stomach immediately after barium meal administration. The caecal score was assigned as follows: 0—no barium; 1—small amount of barium, haustra nearly visible, and organ poorly delineated; 2—medium quantity of barium, haustra visible; 3—large amount of barium, haustra clearly visible, and organ well delineated. *—statistically significant difference (*p* < 0.05).

**Figure 2:**
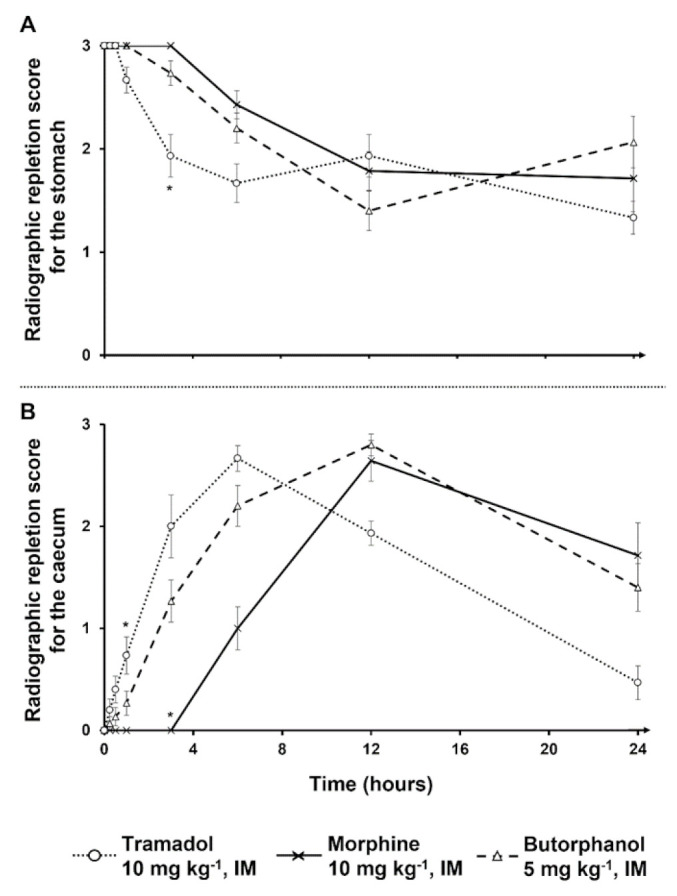
Stomach radiographic repletion scores (**A**) and caecal radiographic repletion scores (**B**), as functions of time. (**A**) Stomach radiographic repletion scores (Mean ± standard error of the mean (SEM)) as a function of time (hours), obtained via a barium follow through examination without and after an intramuscular injection of 5 mg kg^−1^ butorphanol (*n* = 15), 10 mg kg^−1^ morphine (*n* = 14), or 10 mg kg^−1^ tramadol in rabbits (*n* = 15). T0 corresponds to administration of the barium meal. Injection of the opioid was performed 5 min before barium meal administration. The score was assigned as follows: 0—no barium; 1—small amount of barium; 2—medium quantity of barium, heterogeneous repartition of contrast; 3—large amount of barium, homogeneous repartition of contrast or identical to the first radiograph taken at T0 min, when barium has just reached the stomach immediately after barium meal administration. *—statistically significant difference (*p* < 0.05). (**B**) Caecal radiographic repletion scores (Mean ± standard error of the mean (SEM)) as a function of time (hours), obtained via a barium follow through examination without and 5 min after an intramuscular injection of 5 mg kg^−1^ butorphanol (*n* = 15), 10 mg kg^−1^ morphine (*n* = 14), or 10 mg kg^−1^ tramadol in rabbits (*n* = 15). The score was assigned as follows: 0—no barium; 1—small amount of barium, haustra nearly visible, and organ poorly delineated; 2—medium quantity of barium, haustra visible; 3—large amount of barium, haustra clearly visible, and organ well delineated. *—statistically significant difference (*p* < 0.05).

Results of the mixed repeated models indicated effects of treatment, time, and their interaction were significantly different from null, with the exception of the treatment on stomach RRS and interaction terms for both contraction counts. The different treatments effects on stomach and caecum RRS are summarized in Table 1 and Figure 1 and Figure 2.

Considering the whole 24 h timing, no opioid treatment led to significative differences between the stomach means. On the contrary, caecum means were significantly different with morphine and butorphanol treatment (Table 1).

However, comparing radiographic individual time points separately revealed significative differences between treatments. Indeed, radiographic caecum mean RRS of rabbits with butorphanol treatment were significantly different from their control at 30 min and 1 h, but no differences were found affecting the stomach mean RRS (Figure 1A). Mean of stomach RRS were significantly different 3 h and 6 h with morphine treatment (Figure 1B). Caecum mean RRS of rabbits treated with morphine were significantly different from control rabbits at 30 min, 1 h, and 3 h (Figure 1B). Tramadol had no significant effect either on the stomach mean RRS or on the caecum mean RRS (Figure 1C).

As the time elapses, stomach mean RRS is supposed to decrease. However, in the butorphanol-treated rabbits (Figure 1A) at 24 h, and in tramadol-treated and control rabbit (Figure 1C) groups at 12 h, the stomach mean RRS increased instead of continuing to decrease, although no additional barium was intentionally administered to the rabbits.

Stomach mean RRS were not significantly different in all treated rabbits except at 3 h with morphine and butorphanol treatments, where there was a significant difference among treatments (Figure 2A). Indeed, at 3 h with morphine or butorphanol treatment, the respective rabbit stomach mean RRS was different in comparison with the stomach mean RRS of tramadol-treated rabbits. With regard to the comparison of caecum mean RRS with butorphanol treatment and tramadol treatment, no significant difference was found (Figure 2B). Morphine treatment led to significant differences at 1 h and 3 h injection in comparison with tramadol treatment (Figure 2B).

Finally, opioid treatment did not lead to significative effects on pyloric or duodenal contractions.

## 4. Discussion

The findings of the present study suggest that a single dose of an opioid can affect gastrointestinal transit in healthy rabbits, and this effect seems to be temporary. Indeed, significant differences were found at some time points during radiographic examinations, but barium meal continued its physiological progression. While morphine and butorphanol temporarily significantly slowed down progression of the alimentary bolus, no effect was observed after tramadol treatment. A single dose of an opioid did not affect local gastrointestinal contraction, neither on the pylorus nor the duodenum. Among all the three opioids studied, tramadol had the least effects on physiological gastrointestinal motility.

Contrary to another study published about buprenorphine effects on the gastrointestinal motility [4], no evidence of any local effect on the pylorus or the duodenum contraction were found. The differences might be explained by the different statistics method used and expression of results. Moreover, those differences might also be explained by the different pharmacological action of the opioids tested. Indeed, morphine is a pure µ agonist, butorphanol is a mixed agonist/antagonist molecule [13], and tramadol is a µ agonist molecule [14].

Time-lapse analysis of mean RRS at the stomach and caecum levels allows assessment of alimentary bolus kinetics and highlights physiological progression in control animals compared with treated rabbits. Progression of the alimentary bolus in the stomach was delayed 3 h with morphine administration. Consequently, caecum filling and emptying were also delayed. While caecum emptying began at 12 h with morphine and butorphanol treatment, it already began at 6 h in the control and tramadol groups. These findings suggest that morphine and butorphanol cause food retention in the anterior part of the digestive tract. In addition, morphine is known to inhibit small intestine contractility in rabbits in vitro [15].

Stomach filling can reflect colonic motility in the rabbit. Indeed, caecotrophy is a behavioural and physiological characteristic of rabbits that has not been described in any other domestic animal so far [16]. A colonic separation mechanism known as ‘wash-back’ [17] results in the production of two different types of faeces: hard faeces, which are composed of large undigested fibres, and soft faeces, which are called caecotrophs and are composed of fermentable small fibres. Caecotrophs are processed in the caecum and are rich in vitamin B, lysozymes, and micro-organisms. They are ingested without being chewed, directly from the anus [16]. In the present study, ingestion of barium-filled caecotrophs might have increased the stomach mean RRS for the tramadol-treated rabbits at 12 h and the butorphanol-treated rabbits at 24 h. Based on our results, tramadol could be considered as a gut motility, non-interfering opioid, at least in rabbits and at 10 mg kg^−1^, unlike other opioids in the horse [11]. Based on the absence of any increase in the stomach mean RRS at 12 h, because of the absence of ingested caecotrophs, morphine would act at the level of colon. Unexpectedly, no caecotrophs were observed in the butorphanol control rabbits. It may be hypothesized that either animals were disturbed during the caecotrophy period or that the amount of caecotrophs eaten was not enough to be highlighted by the statistical model.

Morphine analgesic activity in rabbits has a reported duration of 4 h [18] while butorphanol has reported half-lives, in rabbits, that varies between 1.64 h if administered via IV and 3.16 h if administered subcutaneously [19]. Based on the radiographic findings of the present study, the duration of action of the investigated substances can be extrapolated. Indeed, caecum mean RRS for butorphanol-treated rabbits are significantly different from controls between 30 min and 1 h, whereas caecum mean RRS for morphine-treated rabbits are significantly different from controls between 30 min and 3 h. Twenty-four hours later, no differences were statistically demonstrated neither for morphine nor for butorphanol-treated rabbits. These observations also demonstrate that gut slowdown is temporary, and that food content goes on moving through the gut.

In a recent study [20], ultrasonographic examination of intestinal and stomach contractions in rabbits were evaluated using ultrasonographic B mode and pulsed wave Doppler methods. It was concluded that the high agreement between the two methods at the duodenum and jejunum levels allows using B-mode instead of duplex Doppler to evaluate intestinal motility. In the present study, no significant effect of opioid treatment was found either on the mean of pyloric contraction counts or on the mean of duodenal contraction counts. However, a temporary slowdown of the alimentary content at the level of the caecum in rabbits treated with morphine and butorphanol was found with barium meals. This implies that barium meals slowed between the stomach and the caecum, probably because caecum filling was delayed. Therefore, based on our results, we cannot confirm whether the pyloric or duodenal contractions were efficient nor that their counts reflect local gastrointestinal motility.

Overall motility of the gastrointestinal tract in tramadol-treated rabbits did not differ from motility in control animals, whatever the parameters measured. So, tramadol seems to be a safe drug for rabbits, at the dose tested in the present study, at least if gastrointestinal transit is considered. This finding is in agreement with observation in humans, in whom tramadol has less gastrointestinal effect than codeine [21] and less gastrointestinal side effects on the gut as a whole, even though minor colonic effects are sometimes observed [22]. This lack of gastrointestinal effect is confirmed in isolated guinea pig distal small intestine [23]. In rabbits, no studies report adverse effect of tramadol on the gastrointestinal transit. One study performed in horses pointed out the tramadol potential gastrointestinal side effects. However, this study dealt with multiple administrations of tramadol [24].

Drug dosages that were used are probably not equipotent [25], so their analgesic properties cannot be compared, which is a limitation of this study. Equipotency or equianalgesic ratio of an opioid, often used in human medicine, is the ability to produce the same analgesic level than morphine. Nonetheless, dosages cannot be extrapolated from human medicine because opioid equipotency is not known in the rabbit. The main focus of the present study was on the gastrointestinal side effects of opioids in rabbits, and the aim of the protocol was to maximise the probability of measurable gastrointestinal side effects. To support this objective, maximal dosages of morphine, butorphanol, and tramadol were retrieved from a drug formulary book for exotic pets [26]. Few studies have reported the analgesic efficacy of opioids in rabbits [3,13,18,27]. It cannot be demonstrated that the dosages for all drugs except butorphanol and morphine had analgesic properties at those dosages, given no analgesiometric study was performed.

In clinical practice, morphine is used at a much lower dosage that ranges from 0.5 mg kg^−1^ to 2 mg kg^−1^ [28] but only 3 mg kg^−1^ of morphine has been proved to be efficient in a rabbit model of thermal threshold analgesiometric study [18]. In humans and mice, the dose required for analgesia is higher than the dose that causes constipation [29]. Thus, the slowing effects of morphine on gastrointestinal transit in the present study might support the latter information applied to rabbits, given that 10 mg kg^−1^ is higher than 3 mg kg^−1^. In the authors’ practices, butorphanol is often used with a benzodiazepine, such as midazolam, for rabbit sedations. Doses range from 0.5 mg kg^−1^ to 1 mg kg^−1^, which is also supported by other authors [30,31,32]. Tramadol is an opioid which has been rarely studied in the rabbit. The maximal dosage reported is from a pharmacokinetics study, which states that the dose orally administered to rabbits did not achieve consistent plasma levels responsible for analgesia in humans [14]. However, no analgesiometric study was performed confirming the absence of analgesia. Clinically, tramadol is often used at 10 mg kg^−1^ [33].

Although main focus of this study was the opioid treatment effect on the gastrointestinal motility, it may be possible that other molecules could have interfered with gastrointestinal tract. The nasogastric tube placement was performed 24 h before the radiographic series and it could be hypothesised that all drugs needed for the tube placement (i.e., medetomidine and lidocaine) were not washed out. Medetomidine is an alpha-2-agonist molecule often used in rabbit anaesthesia. Its use in conjunction with ketamine has been postulated to be responsible for perianaesthetic mortality in rabbits and with post anaesthetic gastrointestinal complications [5,34] but also with potential gastrointestinal side effects [35]. Lidocaine, on the contrary, has been proven to elicit higher gastrointestinal motility in rabbits following ovariohysterectomy when administered through an intravenous constant rate infusion [3]. It is unknown if a nasal administration could have a similar effect on the rabbit gastrointestinal tract. Nasogastric tubing could also have interfered with the rabbit transit because it could have led to a decrease in food consumption. However, during the experiment, no appetite differences were detected when rabbits got their nasogastric tubes. In order to increase the comfort of the animals, tubes were removed one hour after the opioid injection, so animals did not keep the nasal device during the whole experiment. No significant epistaxis was noted, neither any sneezing nor any nasal discharge.

All the animals were male rabbits, in order to standardize the sample and to avoid gender effect. Indeed, it is known that sex could interfere with the pharmacokinetics of opioids in humans [36] and animals [37]. Consequently, the sample studied is not representative of the whole rabbit population and it could be another limitation of the study. Group housing of sexually mature animals can lead to fights among individuals and stress, which can induce a decrease in food consumption and even a gastrointestinal stasis. To minimize the risk of conflicts, multiple feeding points were created in the enclosures and the rabbits were provided 0.6 m² per animal. Total protocol duration did not last more than 3 weeks, so rabbits ended the experiment at 4 months old.

## 5. Conclusions

In conclusion, the present study suggests that a single IM injection of 10 mg kg^−1^ tramadol does not modify the digestive transit time in healthy rabbits. Tramadol appears to have less colonic adverse effects because it preserves the normal caecotroph production cycle. In contrast, a single IM injection of 10 mg kg^−1^ morphine or 5 mg kg^−1^ butorphanol temporarily slows down gastrointestinal transit and prevents physiological progression of the alimentary bolus without inducing ileus. As opposed to data in the literature, where chronic opioid use leads to constipation in humans [1], our data show that alimentary bolus progression can be slowed down after a single administration of morphine or butorphanol in rabbits.

## Figures and Tables

**Table 1:** Least square means (and standard errors) for each treatment (butorphanol 5 mg kg^−1^, IM; morphine 10 mg kg^−1^, IM; tramadol 10 mg kg^−1^, IM), on stomach and caecum radiographic repletion scores.

	B	M	T
Stomach RRS (lsm)	−0.23 ± 0.07	−0.29 ± 0.07	0.05 ± 0.07
Caecum RRS (lsm)	4.08± 0.79 ^a^	7.97 ± 0.83 ^a^	1.22 ± 0.79

B—butorphanol; M—morphine; T—tramadol; RRS—radiographic repletion score; lsm—least square means. ^a^  *p*-value < 0.05.

## Data Availability

The data presented in this study are available on request from the corresponding author.
